# Supporting Iraqi Kurdistan Health Authorities in Post-conflict Recovery: The Development of a Health Monitoring System

**DOI:** 10.3389/fpubh.2020.00007

**Published:** 2020-01-30

**Authors:** Leonardo Emberti Gialloreti, Faiq B. Basa, Stefania Moramarco, Adil O. Salih, Haveen H. Alsilefanee, Sivar A. Qadir, Antonia Bezenchek, Francesca Incardona, Daniele Di Giovanni, Revan Khorany, Luma H. H. Alhanabadi, Shahla O. Salih, Gorgees S. Akhshirsh, Bayar S. Azeez, Berivan A. Tofiq, Leonardo Palombi

**Affiliations:** ^1^Department of Biomedicine and Prevention, University of Rome Tor Vergata, Rome, Italy; ^2^Rizgary Teaching Hospital, Erbil, Iraq; ^3^Directorate of Health, Sulaimaniya, Iraq; ^4^Family Medicine, Directorate of Health, Duhok, Iraq; ^5^Department of Biomedicine and Prevention, University of Rome Tor Vergata, Rome, Italy; ^6^Informa-PRO, Rome, Italy; ^7^EuResist Network, Rome, Italy; ^8^Department of Industrial Engineering, University of Rome Tor Vergata, Rome, Italy; ^9^EuResist Network, Erbil, Iraq; ^10^Primary Health Care Department, Preventive Health Affairs Directorate, Duhok, Iraq; ^11^Department of Statistics and Informatics, University of Sulaimaniya, Sulaimaniya, Iraq; ^12^Department of Civil Engineering and Computer Science Engineering, University of Rome Tor Vergata, Rome, Italy; ^13^Computer Systems Engineering, Erbil, Iraq; ^14^Directorate of Health, Halabja, Iraq

**Keywords:** epidemiological surveillance, e-health system, ICD-10, post-conflict, public health, Iraq

## Abstract

**Background:** Iraq has endured several conflicts and socio-political tensions that have disrupted its public health system. Nowadays, because health data are not collected on a routine basis, the country still lacks proper statistics and, consequently, response plans to meet present and future health needs of its population. An international partnership is developing in the Iraqi Kurdistan a Health Monitoring System with the aim of supporting evidence-based health policy decisions.

**Methods:** The pilot phase for assessing the feasibility of the programme was launched in 2015. In 2018 the implementation phase began. The first step was to choose the software platform and the coding system, as well as to identify the public hospitals (PH) and Public Health Centers (PHC) to be included in the e-health system. The technical infrastructure of each PHC or PH was updated. The staff of each center was trained in the use of the e-health system and in disease coding. Several seminars introduced regional and district health managers to the basic concepts of data-driven decision making. A local team of experts was trained to create a highly specialized staff with the objective of “training the trainers” and ensuring the future self-sufficiency of the system.

**Results:** By September 2019, 59 PHC and PH were entering data in the Health Monitoring System, while 258 health operators (medical doctors, administrative staff, nurses, statisticians, IT and public health specialists, pharmacists) have been already trained. Currently, more than 600,000 disease events have been collected. Additionally, further 734 medical doctors, statisticians, and health managers have been trained on the basics of public health practice. The goal during the next 3 years is to reach 120 operative centers within the region, envisaging a subsequent expansion of the system to all Iraq.

**Conclusions:** The creation of a functioning health monitoring system is feasible also in regions characterized by socio-political tensions. However, multiple stakeholder partnerships are essential. The provision of an e-health information system, coupled with the establishment of a team of local experts, allows the routinely and timely collection of health information, facilitating prompt responses to present and emerging needs, while guiding the formulation and evaluation of health policies.

## Introduction

Many countries are struggling with the legacies of armed conflicts. As has been highlighted in the literature, such post-conflict scenarios are often characterized by “multiple transition processes” (1). The Middle East (where the human toll of old and new violent conflicts has been enormous) is in an unprecedented state of flux. The events of recent decades have had a profound impact on the public health systems of many countries and, therefore, on the health of their populations (2, 3). Basic infrastructure has been destroyed, health structures have collapsed, primary healthcare has been disrupted, health professionals have been killed or forced to flee, and resources have been diverted from public investment to belligerent purposes: all of these conditions are contributing both to an increased number of casualties and to the long-term health instability and frailty of these countries (4). Regions that have experienced armed conflicts, especially when those conflicts are recurrent and protracted, face enormous challenges in recovering their health systems (4). Devastating consequences, which arise as both direct and indirect effects, may even last for years after conflicts end (5). In fact, while the phase of responding to an emergency can be relatively short, the recovery phase is a complex and potentially years-long process that involves many entities and participants, as well as multiple investments and resources.

### Background on Iraq

During the 1970s, Iraq was considered to have one of the most advanced health systems in the Middle East (6). However, since the late 1980s, the country has experienced a progressive and steady decline due to recurrent international and communal armed conflicts and fighting, sometimes related to alleged or actual chemical, biological, radiological, and nuclear (CBRN) threats (7). In 2014, the rise of the so-called Islamic State (ISIS), its occupation of vast regions of the country, and the subsequent battles to retake control of those areas, worsened the conditions of Iraq's already debilitated health system (8, 9). Different dimensions of the country's healthcare assets, from access to health services (10) to food security (11), suffered. All of this resulted in an increase in mortality and morbidity, as well as in vast population displacements to the Iraqi Kurdistan Region (KRI), an autonomous region of Iraq and a relatively safe area in which to seek refuge (12).

Despite the defeat of ISIS in 2017 and the overall economic and institutional improvements in Iraq, the country continues to experience instability; there are multiple reasons for its high levels of fragility and poor resilience. According to the Fragile States Index (FSI) for 2019, Iraq remains in a situation of high alert, meaning that it continues to be considered unstable and could easily regress and lose its recent gains (13). In fact, despite the military defeat of ISIS, some cells of this organization remain active in different areas of Iraq. According to data from the United Nations Assistance Mission in Iraq (UNAMI), 939 civilians were killed in acts of terrorism and in episodes of violence during 2018 (14). In 2019, the UN Refugee Agency identified more than six million people of concern in the country (15) and 1.7 million internally displaced people (IDPs) (16). In terms of areas of displacement, of all Iraqi regions, the KRI now hosts the largest numbers of protracted IDPs (30%) (17) and refugees (99% of the 250,000 Syrian refugees in Iraq live in the KRI) (15), putting even more pressure on an already debilitated public health system (18).

Particularly, Iraq and its Kurdistan region are suffering from what has been defined as the “single most critical failure of development over the past 30 years” (19), that is, the lack of birth, disease, and cause-of-death data systems. This situation is mainly due to the limited availability of health-related and epidemiologic information, with available data usually being of uncertain quality. However, reliable information on diseases and deaths in the population should be the foundation of any health system. The consequence of the lack of such a system is that health policies are developed based on poor evidence (20). In fact, in the KRI, as in all Iraq, there is limited documented knowledge about the actual health situation of the population; information is not routinely collected and used for health planning, and the impact of the provided care is not efficiently monitored or evaluated (21, 22). These limits do not allow for the full range of analyses that politicians and managers would need to guide health policies or monitor health services and/or results. Therefore, much effort is still needed in Iraq to guarantee a well-functioning public health system (23).

### Setting Up an Epidemiological Surveillance System in Iraqi Kurdistan

Considering this context, the University of Rome Tor Vergata and the Ministry of Health of the Iraqi Kurdistan Region, with the financial support of the Ministry of Foreign Affairs and International Cooperation of Italy, set up a project in 2015 called “Development and implementation of a health monitoring and epidemiological surveillance system in Iraqi Kurdistan.” The project's mission is to support Iraq during the post-conflict phase of recovery by laying the foundations of an efficient health system that can provide high-quality essential services to the population. This joint project is establishing the first Health Information System in the KRI and in Iraq, with the purpose of gathering data on health determinants, births, deaths, and health service performance. The main objective is to enable the health authorities to collect, analyse, and interpret health data, based on an International code system, as well as to improve access and quality of public healthcare. These results can be achieved by enhancing health authorities' evidence-based decision-making and health planning processes. Therefore, a crucial element of this project is the development of a public health culture among health personnel, which will be achieved by strengthening their technical skills and their leadership and management capacity. Continuous training of health personnel at all levels is another fundamental feature of the project.

## Materials and Equipment

### Setting

**Figure 1:**
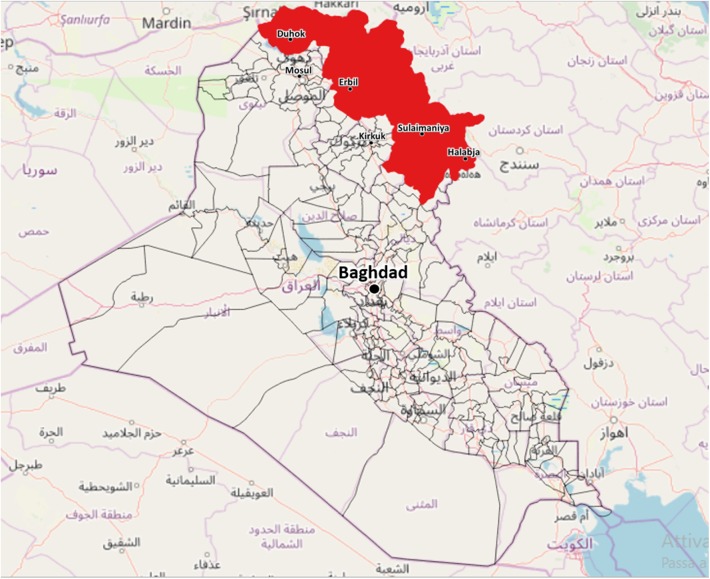
Geolocation of the Iraqi Kurdistan Region (KRI), the work area of the project.

Considering the complex geopolitical situation in Iraq, the KRI was chosen as the site of the pilot phase of the project (Figure 1). The KRI is situated in the north of Iraq, bordered to the west by Syria, to the east by Iran and to the north by Turkey. The region is between 40,000 and 50,000 square kilometers in area and has an estimated resident population (in the absence of census data) of approximately 5.9 million people, consisting of Kurds, Assyrians, Chaldeans, Turkmen, Armenians, and Arabs (24). Overall, 35% of the population is younger than 15 years, 61% belongs to the active age group and 4% is 65 or above. The median age of the population is 19.5 years (25). Administratively, the region is divided into four governorates: Duhok, Erbil, Sulaimaniya, and Halabja. The public health care delivery system of the KRI is basically divided into two levels: the hospital sector and the primary care sector, which is based on primary health centers (PHC). At present, there are 74 hospitals, 249 PHC where a medical doctor is present and 598 PHC managed exclusively by non-medical personnel (9, 26). In 2017, the Kurdistan region spent 4.8% of its GDP on healthcare (26).

### Choice of the Health Information System

As the starting point of the project, it was necessary to identify the appropriate software platform to use in the system. Several platforms were surveyed and analyzed. Finally, we opted for the District Health Information Software 2 (DHIS2), a health management information system platform, already in use by 67 low and middle-income countries. It is an open-source Javascript based platform that allows users to enter data directly from the periphery on the central servers, using only a web browser and even slow or discontinuous Internet connections. When the connection is slow or missing, the entered data are saved temporarily in a local database stored on the browser cache. can DHIS2 can capture data on any type of device, including desktops, laptops, tablets, and smartphones. Another useful feature of DHIS2 is the possibility to generate directly on the same browser session pre-stored aggregated statistics in different formats (pivot tables, charts, maps) or create personalized ones, either modifying those already existing or generating new ones. The presence of a wide community of DHIS2 users and experts in various regions facilitates the sharing of experience with its deployment and implementation, assuring the reliability of the platform (27).

Once DHIS2 had been chosen as a software platform, we decided whether to manage the hosting directly or use outsourcing. Due to the continuous evolution of the DHIS2 software, we decided to rely on a provider with experience hosting DHIS2. The provider manages the software for many other entities and can guarantee installation stability, data security and help in solving problems caused by software updates, which are inevitable due to the changes caused by the evolution of the data structure. We chose BAO (Washington, DC, USA) system as a partner (28), both for the company's skills and experience in hosting DHIS2 installations and for convenience, data safety, security and the possibility of easily scaling up the DHIS2 software.

The health information system is flexible: it began with a pilot project, followed by the creation of a hierarchy of health units (hospitals and PHC) connected with the four general health directorates (Erbil, Duhok, Sulaimaniya, and Halabja), which in their turn relate to a central regional root node based in the Health Ministry of KRI. New units can be added progressively, covering gradually the region. If some health units have specific requirements, there is the possibility to add additional information to the created monitoring programmes. For example, we added *pot hoc* an “Admission Mode” item to the “Hospital Discharge” Programme as it was required only in some Emergency Departments enrolled later in project. Data collected by the other centers was not affected by this programme modification. The health information system was set up in accordance with the Iraqi and Kurdistan Region laws and in cooperation and with the approval of the Ministry of Health of KRI. Such a public health surveillance system was developed following the most updated “WHO Guidelines on Ethical Issues in Public Health Surveillance” (29). In addition, in conformity with Guideline 10 of the mentioned document, identifiable data has been appropriately secured (29).

Considering the characteristics of this public health intervention, the institutional review board of the University of Rome Tor Vergata waived any requirements for further ethical approval and written informed consent at this stage, in accordance with the national legislations and the institutional requirements.

### Training

A key aspect of the development and sustainability of such a project is the training of the personnel who will manage the system itself. Thus, specific workshops and wide-ranging training sessions were held. Healthcare professionals (medical doctors, nurses, and administrative staff) were trained on-site in the use of the health information system, including disease coding, data recording, and presentation. After the first basic training sessions, the staff continued to receive consistent on-the-job training. Additionally, throughout the pilot phase, a team of local trainers was established. The objective of this “training of trainers” was to begin to ensure the self-sufficiency of Iraqi Kurdish personnel in preparing new staff for the use of the health monitoring system, with the goal of guaranteeing the project's continuity and sustainability. Therefore, as part of the project, starting in the academic year 2018–19, the University of Rome Tor Vergata is offering six paid Ph.D. positions related to the project itself: three in “Nursing Sciences and Public Health” and three in “Computer Science, Control and Geoinformation.” The aim of these 3-year-long PhD is to prepare highly specialized staff to direct the entire system once it is under the management of the local authorities. These specialists, together with the hundreds of other operators trained in the centers, directorates, and ministry, will have to guarantee the future continuity and sustainability of the created health monitoring system. By September 2019, the trained health personnel amounts to 258 people: 142 medical doctors, 53 administrative staff, 36 nurses, 12 statisticians, 7 information scientists, 6 public health specialists, and 2 pharmacists. By the end of the project, the trained operators are projected to be about 500.

Another feature of the programme was the establishment of continuous training. In fact, the development and integration of the system, as well as the expansion over time of the programme and of the functions of the system, also made it necessary to set up an online training platform.

With the wider vision of supporting the development of a “culture of data for action” (26), seminars and practical training sessions about health monitoring systems, the overall meaning of epidemiological surveillance, and ICD-10—the Classification of Diseases used to input clinical data in the e-health system—were organized for the health personnel of the region, even if they were not enrolled in the programme at this stage.

## Methods

### Pilot Phase

The pilot phase, during which we assessed the feasibility of the programme, was launched in June 2015. The first step was to identify the hospitals and PHC that could be included in the surveillance system. The following selection criteria were used: 1- willingness to participate; 2- serving a relatively large population; 3- presence of medical staff; 4- location/site and distribution among the different areas of the region. Each PHC or hospital was visited before inclusion in the programme to determine the status of the technical infrastructure, to verify the availability and adequacy of the existing personnel and to make arrangements for subsequent actions. For each center, the personnel were classified according to function, modality of participation, and level of training required. As in KRI there is a limited supply of doctors, but not of nurses and/or of administrative staff, the doctors were mainly trained in disease coding so that they could forward the diagnosis to nurses and administrative personnel trained in data entry, without subtracting time from visiting patients. Wherever possible, data are entered directly in the electronic net. However, in several centers the doctors write the diagnosis on paper, which is then transmitted to the administrative staff for data entry.

No financial incentives were provided to the healthcare workers, since the personnel was specifically assigned by the local health authorities to work for the system. As a non-financial incentive, the workers received—in the framework of an official ceremony—a certificate of merit, recognized by the health authorities as a title that can be possibly used for one's career prospects. The managerial staff of the centers were involved in the definition of the tasks within the programme and in motivating the remaining personnel.

During the pilot phase, the chosen software platform had to be tested and adapted to the local environment. All participating health centers had computers already available; in some cases, when computers were missing, they were provided by the Kurdish health authorities, while the project staff bore the responsibility of hard- and software maintenance. To use the system with devices already available in the health centers, appropriate operating apps had to be developed. In addition, because a few hospitals already had basic informatics systems to record their own data, information collected through these databases needed to be converted for import into the new system. Therefore, new data import software was developed, while allowing the hospitals to continue to use their usual databases.

The testing phase mainly covered two areas: 1- creating data sets with aggregate data entry and 2- developing programmes with disaggregate event data capture. After analyzing the data reports produced by the tests, the project management decided to use programmes without registration, using the “Event Capture” app. This app allows the importation of existing data from hospital databases, mainly using API calls, with no main transformation of the hospital data structure. However, physicians or health technicians can also enter data in real time without having to complete further data aggregation tasks. We decided to begin by capturing only essential data to make the data collection process simpler and more user-friendly. We therefore chose five main programmes for which to collect data: “Births,” “Vaccinations,” “Disease surveillance” (diseases diagnosed at health centers), “Hospital discharges” (diseases diagnosed in hospitals), “Deaths,” which are subdivided in “deaths in hospitals” (with identified cause of death) and in “deaths not in hospitals” (without reliable cause of death).

Data entry takes place at each participating center via an online interface (or offline, in the event of a temporary connection failure) of event-capture by PC, smartphone, or tablet. Health information—disease diagnoses, hospital discharges, registrations of births, deaths, immunizations—can be entered in real-time by physicians or health technicians during a visit or passed on to the office of a data-entry manager who enters it. Data can be stored, retrieved, and analyzed, used to monitor the same health facility across different periods, and used for sharing and comparing health information across hospitals, regions, settings, and countries. Data collected at each individual center then flow to a server on the DHIS2 platform in a space dedicated to the individual center. Each center can recall the entered data at any time. Diagnoses are coded using the WHO International Statistical Classification of Diseases and Related Health Problems, tenth revision (ICD-10) (30), the standard diagnostic tool for epidemiology, health management, and research. This resource defines the universe of diseases, disorders, injuries and other related health conditions, listed in a comprehensive, hierarchical way (31). The system is already prearranged to be integrated with ICD-11, when released.

User interfaces for DHIS2 already existed in English and Arabic. With the help of the DHIS2 community, DHIS2 developers, and a translation team, the DHIS2 user interface was first translated into Kurdish-Sorani, a language spoken by ~6 million people, mainly in northern Iraq. The translation into Kurdish-Sorani presented some technical problems because the language, defined by the ISO 639-3 ckb standard, was missing from the basic Java libraries, which are used for the development of the DHIS2 platform software. To address this problem, we directly asked the Java library development team for support. Some topics, such as the name of the language, are still open.

By the end of the pilot phase (December 2016), 29 health facilities located in both urban and rural areas (16 health centers and 13 hospitals) were networked, running an operational system, which began to routinely collect the diagnoses of all referred patients. During the same period, ~100 additional hospitals and health centers were identified, with the aim of including them in the health monitoring system.

### Implementation Phase

After a thorough technical evaluation of the results of the pilot project, the implementation phase was supposed to start during the second trimester of 2017. However, it had to be temporarily put on hold due to the “Battle of Mosul,” the military offensive of Iraqi, Kurdish, and international forces aimed at retaking the city of Mosul from the so-called “Islamic State,” which had taken control of the city in June 2014 (8, 32). The battle, the world's single largest military operation since the 2003 invasion of Iraq (33), was a cornerstone of the fight against ISIS; the battle ended in July 2017 with the liberation of the city (34). Consequently, it was possible to start the implementation phase of the comprehensive programme only in the second half of 2018, once relative stability was re-established in the region.

As a first step, all 29 centers that had been activated during the pilot project were visited, starting with the nine centers that had interrupted the transmission of data to the central server between the end of 2017 and the beginning of 2018. This interruption occurred because the involved staff had their salaries reduced (like all public employees of the region) by up to 70% due to a political conflict between Iraq's central government and the Kurdistan Regional Government. During 2018, when the situation improved and full salaries were restored, these centers re-began to collect data on a regular basis, becoming the “organization units” of the monitoring and surveillance system.

In November 2018, enrolment began of the next 30 centers to be included in the system. The choice of the new centers to be activated was also dictated by analyzing the population density of the different areas of KRI, as this region has highly variable population density. The new centers—distributed over the four governorates of the region—were located in the areas of greatest density in order to widen the catchment area as much as possible. For each center, the availability and adequacy of the staff were assessed and classified according to their functions, methods of participation and level of training required. The staff then participated in some introductory courses about the technical characteristics and objectives of the project, followed by on-the-job training.

### Routine Data Collection, Information Management, and Monitoring Process

After having chosen the platform for the implementation and deployment of the system, the first challenge was to develop solutions that could work in a short time while satisfying the basic data-collection needs of the regional health system. The evolution of the data structure is actually a continuous process and requires constant attention and updating. Even if the essential data remain the same, at any newly opened PHC or hospital, there are some specific peculiarities that must be considered. In some cases, a specific need at one facility might also become useful for all other centers. For example, the birth of triplets was added only in a successive phase.

**Figure 2:**
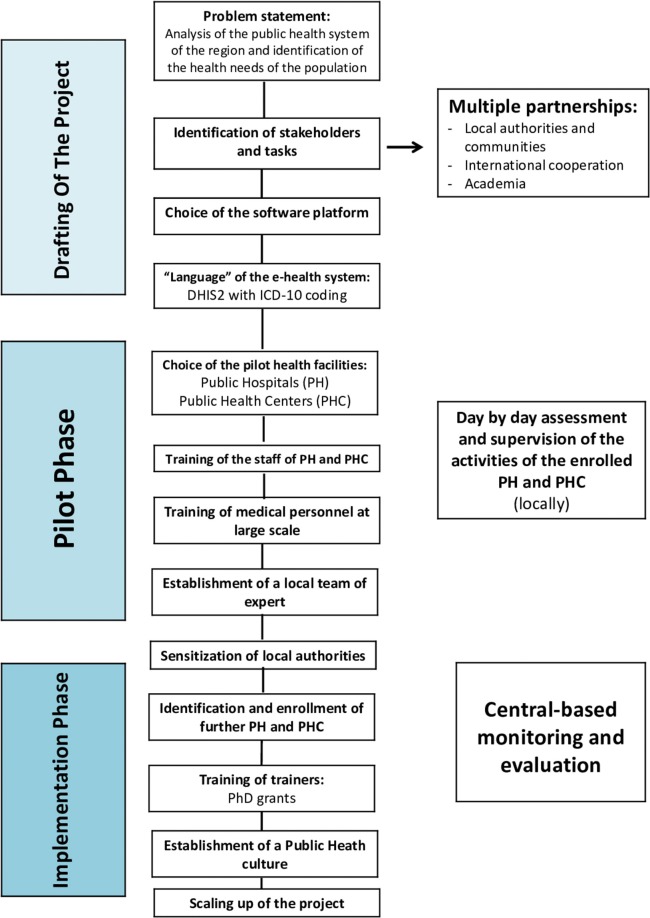
Flowchart of the sequence of steps and decisions needed to set up and run the Health Information System in the Iraqi Kurdistan Region. PH, Public Hospital; PHC, Public Health Center.

The purpose of the system is to store impersonal events data in order to generate aggregate statistics at different levels. Once entered by a “health unit” (hospital or PHC), the data is immediately stored in a central cloud server, using the encryption protocol TLS/SSL and RSA encryption algorithm with a strong encryption public/private key of 2,048 bits. Each health unit has one or more users who are the only authorized persons to enter and visualize the personal data of their patients. Patients are registered by means of a folder number generated by the health unit, which is the only component of the system, which stores sensitive patients' data. This information is not stored in the central system. There are further additional measures to assure anonymization of data: (1) storing in the central system database the patient's age instead of the date of birth; if data are imported form a hospital database or other registry, where a birth date is present, an algorithm calculates the age of the patient at the event date (hospitalization or visit at a health center) and stores it as age in years or months (for children below 1 year) in the central system database. (2) Data stored in the central system database—currently based in Italy, pending a future relocation in KRI—are anonymous, containing just a user file number or ID assigned by the original health unit, gender, age, diagnosis, and some other anonymous additional information that is useful for statistical analyses. This anonymized data is checked by a supervisor team for consistency of the diagnosis with the region and the age range and for other possible incoherencies. Where those inconsistencies exist, they are reported as a feedback to the health center that entered this information. At present, control is still partly automatic and partly human. Communication groups between the operational center and the local staff (using Viber, WhatsApp, and other messaging tools) have been created, resulting in a rapid question/answer mechanism. It is foreseeable that the further expansion of the KRG-HIS system and the inclusion of many more users and large amounts of data will increase the possibility of human errors in data recording or in user and unit management operations. These errors, if not detected and promptly corrected, will continue to be made and could mislead other users. For these reasons, the technical team of the project has to continuously share experiences with the DHIS2 community as well as watch and monitor users' activity in order to detect and correct any error that may arise. As the DHIS2 offers a wide community of developers and users, it is possible to address and fix technical problems relatively quickly. The sequence of steps and decisions needed to set up and run the system are synthetized in Figure 2.

## Results

**Figure 3:**
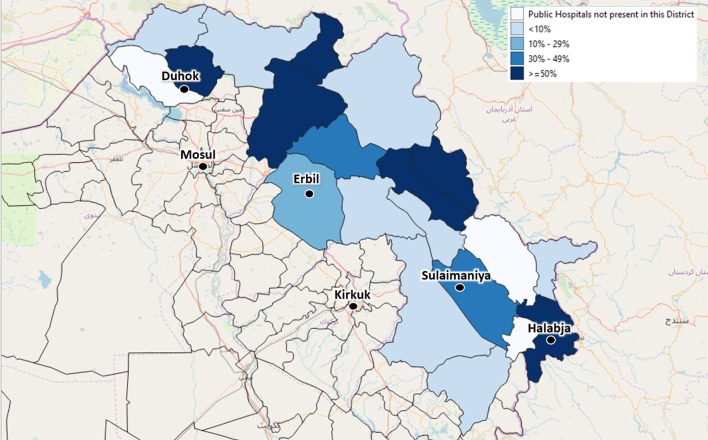
The depicted percentages show the proportion of Public Hospitals out of all available Public Hospitals of each district, which have been already enrolled in the Health Monitoring System by September 2019. The included Public Hospitals have been identified by agreement with the local health authorities.

**Figure 4:**
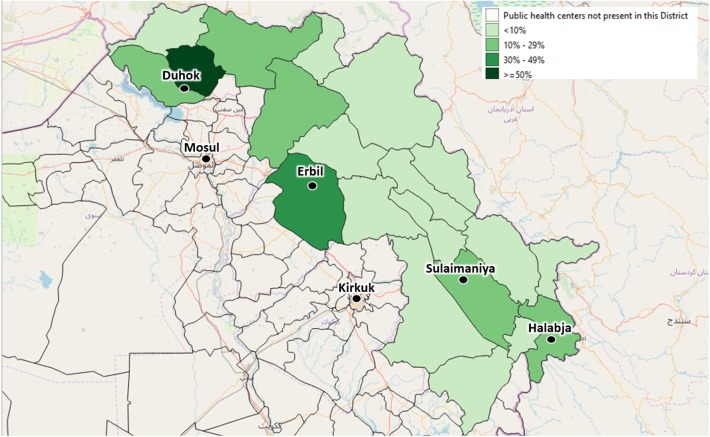
The depicted percentages show the proportion of Primary Health Centers out of all available Primary Health Centers of each district, which have already been enrolled in the Health Monitoring System by September 2019. The included Primary Health Centers have been identified by agreement with the local health authorities.

By September 2019, the centers identified by the local health authorities to be included in the Health Information System were 59 (44 PHC and 15 Hospitals). These centers are evenly distributed across the four governorates, covering different percentages of the total number of centers: 25 are in Duhok governorate, 16 in Erbil, 15 in Sulaimaniya, and 3 in Halabja (Figures 3, 4).

At present, ~400,000 disease events have been collected in the PHC, and ~200,000 in hospitals, making this one of the widest sentinel surveillance systems in the area. For the time being, entered diseases have not been preliminary chosen, but all the diagnoses given daily in the PHC (discharge diagnoses in hospitals) were recorded in the system. Nevertheless, to enable a better understanding of the picture in specific medical areas, some speciality hospitals have been enrolled in the system as well, such as mental health, dermatology, pediatrics, maternity, obstetrics and gynecology, cardiology, ophthalmology, and a hospital for survivors of chemical weapon attacks.

Some of these events have been imported via periodic uploads from existing and ongoing databases, but most have been recorded as direct-event captures by users. This major progress is enabling health professionals to gather statistics on disease diagnoses, hospital discharges, and registrations of births, deaths, and immunizations.

In parallel with the development of the Health Monitoring System, also seminars and practical training sessions were organized with the specific objective to increase the integration, processing and analysis of data among the personnel of the public health care delivery system. By the end of these training courses, the participants had acquired basic skills to develop and implement a protocol for the collection, processing, analysis and presentation of data. They were acquainted with using tables, graphs and/or maps for specific data elements, identifying mechanisms for efficiently detecting data, using internationally recognized classification schemes, managing computer systems for data collection, transferring and transmitting data from the local to the regional level, and using data to reflect on the changes to be made to the health system (“data-driven decision making”). Overall, 734 medical doctors, nurses, statisticians, and public health officials in the region have been trained on these topics.

### The Way Forward

**Figure 5:**
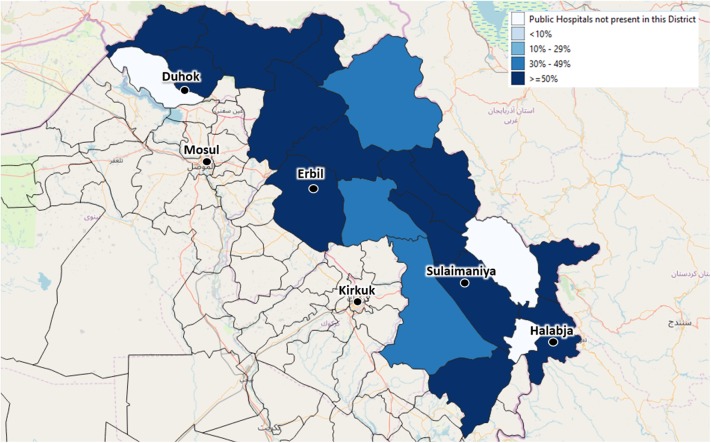
The depicted percentages show the proportion of Public Hospitals out of all available Public Hospitals of each district, which will be enrolled in the Health Monitoring System to ensure the coverage of at least 50% of the Public Hospitals of each area.

**Figure 6:**
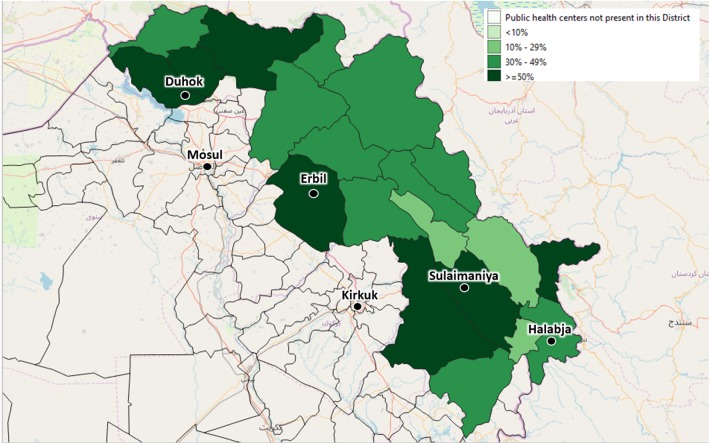
The depicted percentages show the proportion of Primary Health Centers out of all available Primary Health Centers of each district, which will be enrolled in the Health Monitoring System to ensure the coverage of at least 50% of the Primary Health Centers of each area.

At present, data collected by the surveillance system is not fully representative as several centers have not been activated yet. In fact, the overall aim of the project is to ensure the coverage of at least 50% of all primary health centers, family health centers, and public hospitals of KRI by the end of 2021. In order to be actually representative the epidemiological surveillance system will have to include at least 120 main health facilities. Figures 5, 6 show the foreseen scaling-up. Only the integration of the e-health system in all these facilities will allow to build a solid plan for action and to make evidence-based decisions. In addition, contacts with the central authorities of Iraq were initiated, looking toward the future enlargement of the health monitoring and epidemiological surveillance system to the entire Iraqi territory.

During the next 3 years a limited network of health units, particularly hospitals with good laboratory facilities and experienced staff, will be selected as the backbone of a sentinel surveillance system that can be used when high-quality data are needed, so to identify outbreaks and disease trends more rapidly than a wide-ranging but passive surveillance system. At the same time, software and deep-learning algorithms will be developed to enable almost completely automated control. Human intervention will then be limited only to specific situations. Furthermore, in the future, all these activities will be based in Iraq and will be handed over to the local staff and health authorities. The data of several centers (both raw and aggregated) will flow to the health directorate of competence, while the data of the whole region will flow directly to the database of the Ministry of Health of Kurdistan. The statistical staff of the directorates will have access to all the data relating to their province, while the staff of the ministry will have access to the data of the entire region. The statisticians of the health directorates and of the ministry, once trained, will be able to develop all the necessary statistics and produce the relevant reports on a regular basis. Finally, the statistics department of the Ministry of Health will interact with the central Iraqi government and with international subjects in terms of both data exchange and reporting. Beyond the required statistics, which all centers will have to produce, once the staff are trained each individual center will be able to calculate also its own statistics, according to their specific needs.

### Limitations

The primary limitation of this project relates to the fact that in Iraqi Kurdistan, as well as in the whole of Iraq, the state is only just beginning to provide each citizen with a “unique-identity number.” The lack of this identification system makes it difficult to univocally link a specific diagnosis to a given individual. While awaiting the possible future implementation of a unique-identity numbering system, this project adopted the system of event recording. Thus, for the moment, data in the system can be captured only by event and cannot be automatically linked to a specific person. Nevertheless, the software is already prearranged to include unique-identity numbers as soon as they are widely available in the country.

Since the e-health system is becoming the official health information system of the Region, the local health authorities are the guarantee of its sustainability. It is foreseen that the cost of routinely maintaining the system (including training of personnel and salaries of the local experts) should be prioritized by the local authorities. In any case, it has to be highlighted that the system can be run by just reallocating the already existing human resources of the public health service. No new hiring is required. However—like all other aspects of public life—the future commitment of the authorities is strongly interconnected with the wider political and economic assets of the country. Furthermore, as this project is operating in an unstable geopolitical area, both the national and international scenarios will play important roles in the sustainability and implementation of the project throughout the country. This complex picture leaves open the possibility of changing and adjusting the programme's assets according to the country's specific needs.

As it is well-known, the main objective of a surveillance system is to enable health authorities to collect health data, enhancing their evidence-based decision-making. However, at this stage it is too early to be able to evaluate its actual effect on the health of the population. Therefore, results in terms of impact of preventive and curative activities need to be further proven and discussed in future.

## Discussion

Armed conflicts and instable socio-political contexts can severely debilitate health systems, disrupt health services, and hinder access to basic care for large parts of the population (35–37). According to Levy and Sidel (3), it is important not to downplay the adverse effects of conflict on the population's health. Policy makers should also be aware of the indirect consequences of wars and should make the establishment of a health system one of their main priorities after the end of fighting. However, even when battles end, war-torn countries face enormous challenges in health systems recovery because they are likely to remain frail for at least several years. The recovery of a health system is a long-term process whose aim is to revitalize, rebuild, and re-populate/re-use affected areas while moving to self-sufficiency, sustainability and resilience.

A crucial aspect of reconstructing an operational public health care delivery system is the development of health monitoring systems. However, in frail states, it is often very challenging to obtain accurate information on the population's health, as data records are inadequate or data systems are absent, social systems breakdown, and forced migration makes it difficult to collect accurate information. Overall, statistics might come from estimations and projections, which makes them not fully representative of the actual situation of the population.

Regarding Iraq, the significant debilitation of the public health care delivery system due to continuous conflict and fighting reduced the population's health indicators to levels comparable to those of the least developed countries (38). The already compromised healthcare sector, suffering from extensive neglect and damage, experienced another blow following the rise of ISIS (9). At present, even during a relatively peaceful and stable period, huge knowledge gaps about the health impact of wars and terrorism among civilians remain (3). The United Nations Office for the Coordination of Humanitarian Affairs (UN-OCHA) reported in December 2018 that 5.5 million people needed healthcare but lacked access to health services (39). In particular, epidemiological surveillance is almost non-existent. Health data collection is not systematic and standardized. Only a small amount of health data, often sporadic and inaccurate, is available. Furthermore, these are mostly survey data and are not collected on a routine basis through adequate surveillance systems. The methods of routine data collection—besides being mostly paper and non-electronic with information recorded on slips of paper, so that detailed and chronological information on patients is not available—are not standardized across the different provinces. Raw data are rarely available, just as there is little information on data at the district or sub-district level. Furthermore, at present in the KRI, as in all Iraq, there is only limited use of computers, which are often used only for keeping records. In hospitals and health centers, available computers are usually not used for management or surveillance purposes. Likewise, there is a lack of qualified personnel for data entry, processing, and analysis. Data are rarely reviewed or checked, often leading to very low data quality (40). More generally, among the health personnel and authorities, there is a limited “culture of data for action” (26), where data collection, processing, analysis, presentation, and use are routine and relatively easy. In other words, there is a lack of cultural education that uses epidemiological data and translates it into objectives and policies. For all these reasons, data are only rarely used to inform health policy, to plan, or to identify the key issues to be addressed. Moreover, data collected only occasionally cannot be used to measure performance and to develop an efficient quality control system, making it difficult to develop health policies that meet the actual needs of the population. Often, healthcare is still oriented toward short-term objectives, focusing on combating emerging pathologies without systematic planning, prevention, and health promotion. The rapid influx of refugees and displaced persons has added new challenges to health service delivery, further highlighting the urgent need to develop an epidemiological monitoring and surveillance system to intervene effectively in present and future emergencies and to properly plan medium- and long-term strategies to provide high-quality healthcare.

The above needs are even more important if we consider that conflicts are direct and indirect causes of poor population health, which, in turn, increases the risk of a return to conflict. An econometric analysis found a strong association between poor post-conflict population health, measured in terms of infant mortality rate, and war recurrence. A post-war state with an infant mortality rate of 41/1,000 has a 0.5% annual probability of returning to conflict. Such a risk increases by 300% for a rate of 116/1,000 (41). In 2017, the infant mortality rate in Iraq was estimated to be 33/1,000 (42). In other words, the failure to establish adequate health service delivery may increase the risk of a return to conflict. However, to create a health system capable of responding promptly to present and emerging needs and to inform the health policies of the country, one of Iraq's health priorities is to develop adequate epidemiological surveillance and health monitoring systems. Therefore, as a first step in the recovery of the health system, the project “Development and implementation of a health monitoring and epidemiological surveillance system in Iraqi Kurdistan” entails building a system that can provide information on the health situation and needs of the population.

One of the main goals of epidemiological surveillance is to support health management decisions by using solid statistics retrieved from real and consistent data collection systems. As a first step in this complex process, we evaluated the different methods of data collection. There are essentially two main methods: either collecting already aggregated data or collecting all diagnoses individually and then aggregating them. Direct aggregated data collection leads to more compact and readily usable data, but control over the correctness of the data and the possibility to create different statistics is limited (43). The second method, the collection of all individual diagnoses, generates more data and requires more storage and computational power for processing but has several advantages. One important advantage is that the same dataset can be used to generate different levels of aggregation. It is also possible to monitor, in the case of suspicious data, any single event, e.g., when it was created or modified, to guarantee the correctness and integrity of the data. Therefore, we chose this second method.

After decades of conflict, one of the main priorities on Iraq's agenda appears to be the reconstruction of a full-fledged public health system. Reliable, timely, and accurate information on the health of the population plays a paramount role in improving healthcare by enabling effective health policies and efficient practices (20).

Multiple stakeholder partnerships are fundamental to assist the local health authorities in creating a functioning public health monitoring system, essential in guiding the development of appropriate public health interventions. The provision of an e-health information system for epidemiological surveillance, coupled with the establishment of a team of local experts, will allow the routinely and timely collection of accurate health information based on an internationally recognized coding system, thus facilitating prompt responses to present and emerging needs, while improving access to and the quality of public healthcare. In addition, the health information system will guide the formulation and evaluation of health policies, providing useful data for long-term planning and policy development, triggering appropriate and cost-effective interventions, and eventually leading to better health outcomes. Establishing effective health services is a key aspect of a broader development agenda and is of particular importance in a post-conflict environment. Alongside the necessary emergency interventions, a functioning epidemiological surveillance system is paramount to managing complex and fluid health situations in the medium- and long term while building a foundation of preparedness in case of future crises. This project can offer some suggestions and lessons-learned regarding the post-conflict reorganization of the public health systems of other war-torn countries.

## Data Availability Statement

The raw data supporting the conclusions of this article will be made available by the authors, without undue reservation, to any qualified researcher.

## Author Contributions

LEG and LP conceptualized and designed the overall project and the study. LEG, LP, and FB coordinated the activities of the project. FB, AS, RK, and LEG were responsible for site selection and implementation at the field level. AB, FI, GA, BA, DD, and RK were responsible for software generation and management, software implementation, process evaluation, and IT technical assistance. HA, SQ, SS, GA, BA, LA, and BT participated in the programme's implementation, including training of local staff, data collection, and monitoring of the centers. LEG, LP, and FB made and maintained contact with local health authorities. LEG and SM monitored the implementation phase, conducted the research, conceptualized, and wrote the manuscript. AB, FI, and LP contributed to the writing process. All authors reviewed, provided input, suggested adjustments to the manuscript drafts, and approved the final text.

### Conflict of Interest

FI is CEO of EuResist Network. AB is CIO at Informa-PRO. The remaining authors declare that the research was conducted in the absence of any commercial or financial relationships that could be construed as a potential conflict of interest.
